# Historical collections of vascular plants in the Korean Peninsula by three major collectors in the early 20th century: U. J. Faurie, E. J. Taquet and E. H. Wilson

**DOI:** 10.3897/BDJ.9.e66470

**Published:** 2021-06-10

**Authors:** Chin-Sung Chang, Shin Young Kwon, Hui Kim

**Affiliations:** 1 Seoul National University, Seoul, Republic of Korea Seoul National University Seoul Republic of Korea; 2 Mokpo National University, Muan, Republic of Korea Mokpo National University Muan Republic of Korea

**Keywords:** endonym, exonym, U.J. Faurie, georeferencing, herbarium collection, Korea, Quelpaert, E.J. Taquet, E.H. Wilson

## Abstract

**Background:**

The digitisation of historical collections aims to increase global access to scientific artifacts, especially those from currently inaccessible areas. Historical collections from North Korea deposited at foreign herbaria play a fundamental role in biodiversity transformation patterns. However, the biodiversity pattern distribution in this region remains poorly understood given the severe gaps in available geographic species distribution records. Access to a dominant proportion of primary biodiversity data remains difficult for the broader scientific and environmental community. The digitisation of foreign collectors’ botanical collections of around 60,000 specimens from the Korean Peninsula before World War II is ongoing. In this paper, we aim to fill this gap by developing the first comprehensive, open-access database of biodiversity records for the Korean Peninsula. This paper provides a quantitative and general description of the specimens that Urbain Jean Faurie, Emile Joseph Taquet and Ernest Henry Wilson have collected and are kept in several herbaria.

**New information:**

An open-access database of biodiversity records provides a simple guide to georeferencing historical collections. The first set describes E. H. Wilson’s collection of woody plants collected in the Korean Peninsula and preserved at the Harvard University Herbaria (A). This set includes 1,087 records collected from 1917 to 1918. The other collections contain specimens collected by E. J. Taquet (4,727 specimens from Quelpaert (Jeju), 1907–1914) and U. J. Faurie (3,659 specimens from North Korea and Quelpaert, 1901, 1906 and 1907). For each specimen, we recorded the species name, locality indication, collection date, collector, ecology and revision label. This set contains more than 9,400 specimens, with 22% of vascular plants from North Korea and 66% from Quelpaert (Jeju) Island. In these collections, we included some images that correspond to the specimens in this dataset.

## Introduction

Institutions outside the Korean Peninsula hold much of the region’s historical biodiversity information. With nearly 100,000 specimens, including data on specimens stored at foreign herbaria, these institutions have a comprehensive chronological, historical, taxonomic and geographic coverage of Korean plants, including those from inaccessible areas such as North Korea. Despite the abundance of biodiversity information in these collections, there remains a pressing need to make such data accessible and sufficiently integrated to foster query-based enquiries and achieve regional conservation priorities. Creating this open-access database mobilises existing biodiversity information and knowledge within the Korean Peninsula. Through the advantages offered by a database, we could search through historical records of foreign herbaria, generate georeferenced specimen data and produce images of North and South Korean vascular plants. With these goals, the project addressed the imbalance in biodiversity information between South and North Korea and reduced the knowledge gap on the diversity and distribution of vascular plants in the Korean Peninsula.

Historical biodiversity data provide the context for past observations. Here, we present a vascular plant dataset of the Korean Peninsula covering the early 1900s. This dataset consists of three sets: (1) E. H. Wilson’s 1,087 specimens mainly from North Korea from 1917 and 1918; (2) E. J. Taquet’s 4,727 specimens from Quelpaert from 1907 to 1914; and (3) U. J. Faurie’s 3,659 specimens from North Korea and Quelpaert from 1901 to 1907.

These datasets were the first attempts at archive digitisation in both North and South Korea, covering an early period and incorporating data from different sources. The objective was to identify, describe, perform quality control and integrate historical data for the Korean Peninsula into standardised datasets and make them freely available and reliable for end users in terms of fitness for use (Chapman 2005).

## Project description

### Title

Flora of the Korean Peninsula

### Personnel

Chin S. Chang and Hui Kim

### Design description

This project includes specimens conserved in European, American and Japanese herbaria, which this paper identified via the following abbreviations proposed by Index Herbariorum (Thiers 2019): (1) E for the Edinburgh Botanical Garden, (2) P for the Paris Museum Herbarium, (3) LE for the Komarov Botanical Institute of Russian Academy of Science Russia, Saint Petersburg, (4) A for the Harvard University Herbaria, (5) KYO for the Kyoto University Herbarium, (6) TI for the University of Tokyo Herbarium and (7) SNUA for the T.B. Lee Herbarium at Seoul National University. The E (3,017) and TI (1,002) herbaria constitute the largest collections of Taquet, while the KYO (2,714), E (1,475) and P (869) conserve the major collections of Faurie. This is the first attempt by Korean researchers to investigate specimens deposited at various foreign herbaria using a single and uniform protocol. We have visited TI, A, E and KYO, taken photos and recorded them in the database. We have also searched for additional specimens at P, LE and K either from botanical collection papers (Grabovskaya-Borodina et al. 2018) or herbarium websites (Royal Botanic Gardens, Kew 2020, Chagnoux 2020).

### Funding

This research was supported by a BIFA (the Biodiversity Information Fund for Asia) funded by the Ministry of the Environment (BIFA3_14), Government of Japan.

## Sampling methods

### Study extent

Since 1945, the Korean Peninsula has been divided into what are now two countries: North Korea (Democratic People’s Republic of Korea) and South Korea (Republic of Korea). In terms of botanical importance, its notable islands include Jeju Island (Quelpaert) and Ulleung Island (Ulleungdo). Korea’s vascular flora includes 4,831 taxa (Chang et al. 2014) constituting a relevant portion of Eastern Asian flora. There are also plants from three principal biogeographic regions (Chang et al. 2017): (a) the Amur flora, characterised by cold temperate forests and shrubs; (b) the Northern China flora, characterised by deciduous temperate forests; and (c) the China–Japan–Korea (CJK) flora, characterised by warm temperate forests with evergreen forest taxa.

### Sampling description

E. H. Wilson collected 1,200 plant records representing 51 families that he identified with Alfred Rehder (Briggs 1993). These data describe a specimen dataset of Korean Peninsula woody plants preserved at the Harvard University Herbaria (A). E. H. Wilson visited Korea twice: first in 1917 to Oo-rong-do Island (Ulleung or Degelet Island), Quelpaert Island, Mt. Chiri-san, Pingyang, Keijyo (Seoul), Koryo, north-eastern Korea and Mt. Konggo-san and second, in 1918, he revisited the same provinces. Many books and articles on this collector have been published; however, his botanical itineraries have not been well documented (Kim et al. 2010). Meanwhile, Fr. U. J. Faurie made three extended collecting trips to Korea: the central region in 1901, the central and southern regions in 1906 and Quelpaert Island in 1907 (Hayata 1916, Kakuta 1992, Chang et al. 2004). Fr. E. Taquet, who stayed on Quelpaert Island as a Catholic missionary from 1902 to 1915 (Chang et al. 2015), made extensive collections of vascular plants from the Island (Jeju) from 1907 to 1914.

### Quality control

Both Faurie and Taquet did not number their collections chronologically, based on their collecting activities. They seem to have sorted the collections by genera and they assigned numbers to the taxonomic bundles of dried plants. Some of the collection data, such as locality, date or collection number, were missing. The first set of specimens is at E or P, except for some families. Duplicate specimens were widely distributed and could be found at BM, TI, KYO, A, LE and B. Faurie’s collection of several thousand herbarium specimens is deposited in Paris, with duplicates at the University of Kyoto, the British Museum, Kew and elsewhere (Kitagawa 1979, Koidzumi 1936).

Georeferencing: A wide range of historically used toponyms in Korea have Chinese-character origins and can, therefore, be written the same way (Choo 2016, Tanabe and Watanabe 2014). As a result of 36 years of Japanese colonial occupation, Korean place names used for plant collections have become a toponymic enigma. In many Asian countries, Japanese exonyms are names of places in the Japanese language that differ from those given in their dominant language. Japanese botanists or field guides often transliterated these toponyms into the Japanese pronunciation. This has produced many unresolved botanical exonyms, which have been only found on herbarium labels. These Japanese terms for some place names are now a mystery either because they are quite different from endonyms or because of some other obscure etymology. We have prepared a multilingual gazetteer to resolve the inconsistencies, uncertainties and confusion on botanical exonyms in the Korean Peninsula that foreign explorers and botanical collectors in Korea have used over the past 120 years (Table 1, Chang et al. 2015).

**Figure 1.**
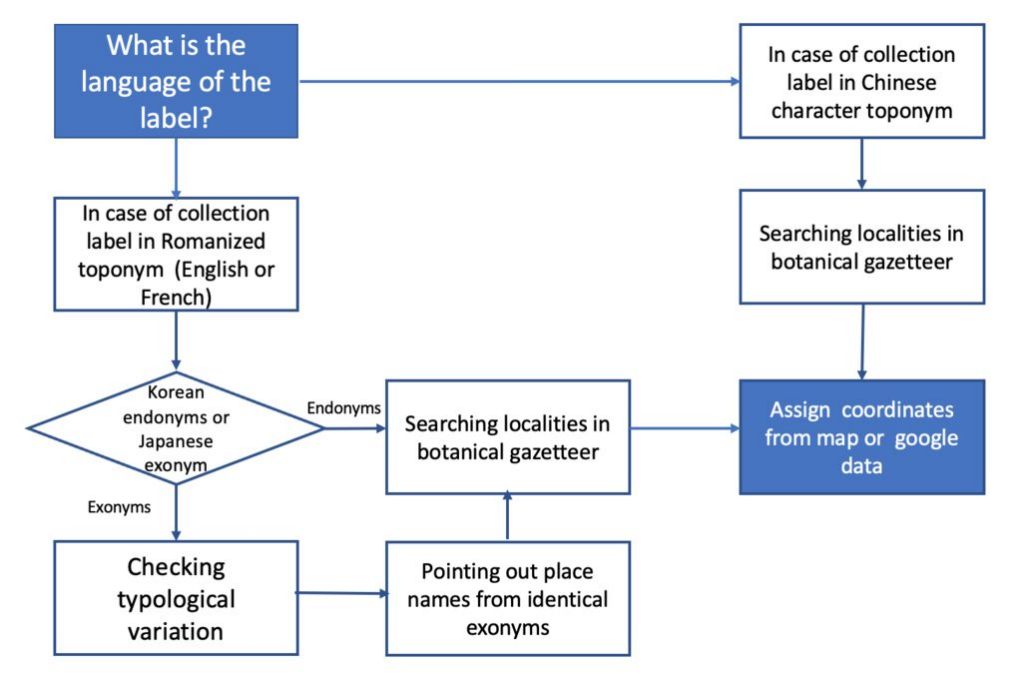
Flow diagram on how to approach labels in different languages and endonyms as well as exonyms.

After the identification of place names, the next step is providing a precise coordination to a biological collection. We always aimed for accurate georeferencing for location coordinates, but sometimes this was not possible because of insufficient information in the place names. Thus, in these situations, we used higher geographic area coordinates, such as counties or cities. To minimise errors, enhance data consistency and maintain integrity throughout the georeferencing process, we modified a procedure adopted by the Chinese type collection project (Fig. 1, Lohonya et al. 2020).

Using the BRAHMS system, we set up a database of herbarium records. We compared the geographic queries with the label information for each specimen to resolve geographic information. We detected and corrected two types of errors: typographical errors and erroneously identified records. After updating the database with recent publications and cleaning the data, we obtained the clear collection data that corresponds to this dataset.

Finally, we generated the Darwin Core Archive to incorporate the metadata in this file and published the data on GBIF, using the Integrated Publishing Toolkit.

## Geographic coverage

### Description

**Figure 2.**
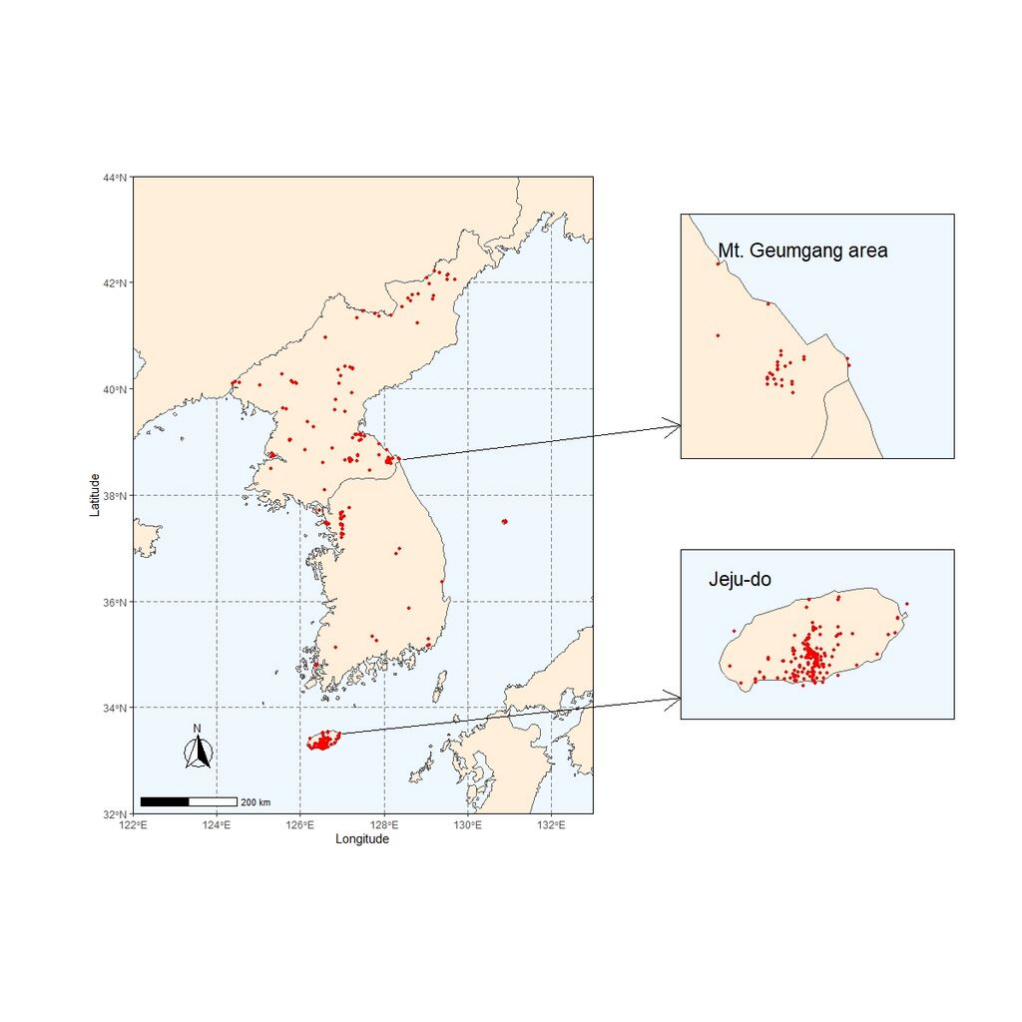
The total occurrence points of vascular plant specimens were collected by three collectors in the Korean Peninsula. Enlarged maps are shown for the Mt. Konggo-san and Quelpaert, where Wilson, Faurie and Taquet made extensive collections.

The Korean Peninsula is located in northeast Asia, between China and Japan. To the northwest, the Amnok River separates Korea from Liaoning Province in northern China and to the northeast, the Duman River separates Korea from Jirin Province in northern China and Far Eastern Russia. Excluding the islands, the Peninsula area covers about 220,847 km^2^. The eastern and northern parts of the Peninsula are characterised by the high mountains. The highest point of the Korean Peninsula is located at Mount Paektu (2,744 m a.s.l.; 41°59N; 128°04E) and stands on the border with China (Fig. 2). The southern area of the Peninsula begins at the Island Marado (33°06N; 126°16E)) at the south of Jeju Island and stretches in an eastwards direction to the islets of Dokdo (37°14N; 131°52E).

### Coordinates

33°06'45.0"N and 43°00'42.3"N Latitude; 124°13'22.8"E and 131°52'22.5"E Longitude.

## Taxonomic coverage

### Description

**Figure 3.**
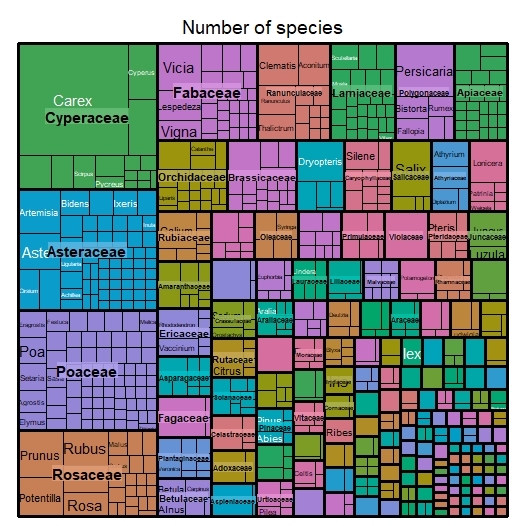
Taxonomic distribution of occurrences amongst vascular plant families in the dataset. The figure was prepared with the “treemap” package in R (Tennekes 2014).

The majority of specimens belong to class Magnoliopsida (6,314 specimens) and Liliopsida (2,198), followed by Filicopsida (765), Lycopodiopsida (33), Equisetopsida (4), Coniferophyta (144) and Psilopsida (1). Our dataset represents 165 families (Fig. 3), of which 16.8% and 11.4% of the specimens belong to the monocot families Poaceae and Cyperaceae and the dicot families Rosaceae and Asteraceae, respectively, followed by Fabaceae (3.9%), Ranunculaceae (3.1%), Apiaceae (2.5%), Dryopteridaceae (2.5%), Polygonaceae (2.3%), Lamiaceae (2.2%), Liliaceae (2.2%), Caprifoliaceae (2.1%) and Fagaceae (1.9%). It further includes 755 genera, with the significant ones being *Carex* (543), *Quercus* (151), *Persicaria* (147), *Dryopteris* (133), *Rubus* (116), *Prunus* (107), *Euonymus* (100), *Salix* (91), *Acer* (91), *Viola* (86), *Thelypteris* (80), *Vicia* (79), *Clematis* (78), *Aster* (77), *Viburnum* (77), *Lonicera* (77), *Ranunculus* (73), *Lespedeza* (70), *Fimbristylis* (66), *Cyperus* (63), *Elaeagnus* (59), *Athyrium* (56), *Adenophora* (55) and *Setaria* (54).

### Taxa included

**Table:** 

Rank	Scientific Name	
phylum	Trachaeophyta	

## Temporal coverage

### Notes

1901 through 1919

## Usage licence

### Usage licence

Creative Commons Public Domain Waiver (CC-Zero)

## Data resources

### Data package title

Flora of the Korean Peninsula

### Resource link

https://www.gbif.org/dataset/65bdd8e3-a27b-4b88-998d-dfb27d528206

### Alternative identifiers

https://kbif.naris.go.kr/ipt/resource?r=flora_korean_peninsula

### Number of data sets

1

### Data set 1.

#### Data set name

Flora of the Korean Peninsula

#### Data format

Darwin Core Archive

#### Number of columns

37

#### Download URL

https://www.gbif.org/dataset/65bdd8e3-a27b-4b88-998d-dfb27d528206

#### Description

**Figure 4.**
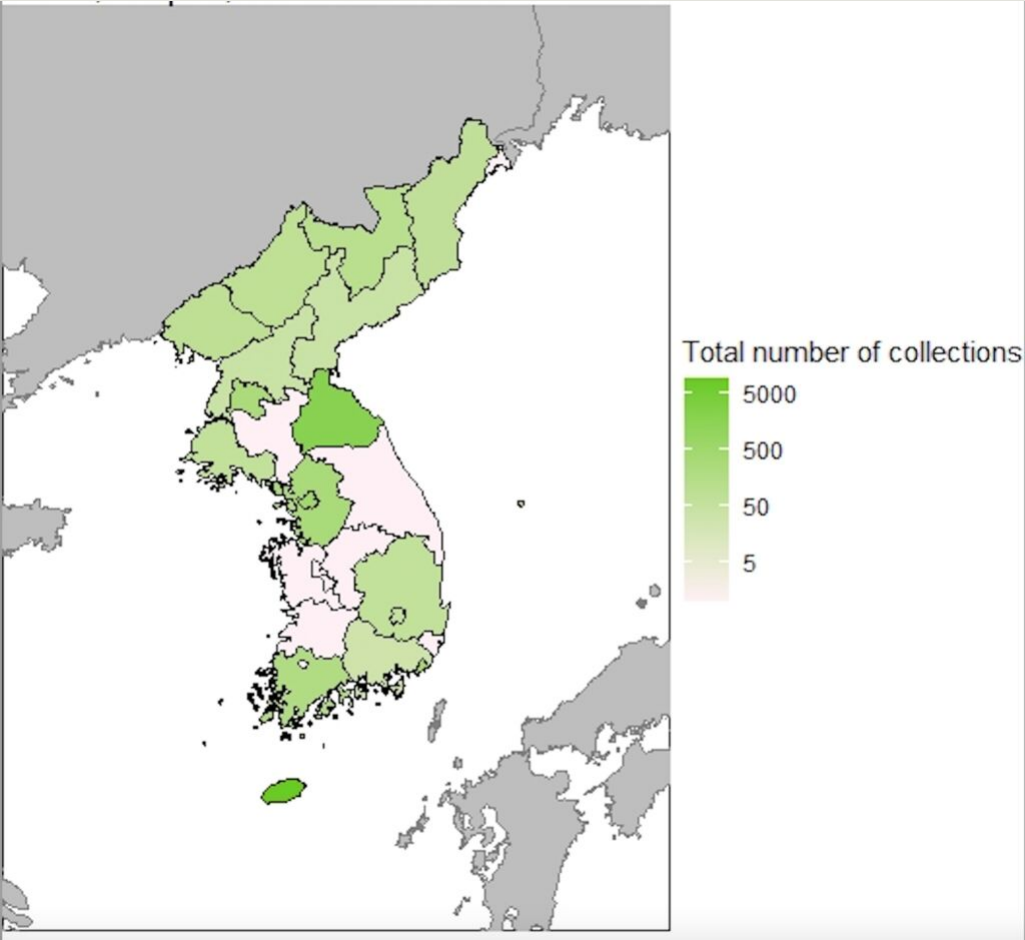
Choropleth map showing the relative frequency of three explorer’s specimens collected from Korean Provinces.

The total dataset contained about 13,981 herbarium specimens including duplicates deposited at several foreign herbaria and gathered by three collectors (U. J. Faurie, E. J. Taquet and E. H. Wilson) from the Korean Peninsula in the early 1900s (Chang and Kim 2020). Through the BIFA project, we actively stored these historical collection data in 2018. All the specimens were stored at various foreign herbaria (A, E, KYO, TI, P, LE and SNUA) and were part of three independent collections; hence, we highlighted the historical collections of E. H. Wilson from 1917 to 1918, U. J. Faurie from 1901 to 1907 and E. Taquet from 1907 to 1914 with type specimens (Fig. 4).

This study presented the digitised data of all these vascular plant collections (1901–1918). The dataset was associated with image collections of plant specimens.

**Data set 1.** 

Column label	Column description
type	The nature or genre of the resource.
institutionCode	The name (or acronym) in use by the institution having custody of the object(s) or information referred to in the record.
basisOfRecord	The specific nature of the data record.
occurrenceID	An identifier for the Occurrence (as opposed to a particular digital record of the occurrence). In the absence of a persistent global unique identifier, construct one from a combination of identifiers in the record that will most closely make the occurrenceID globally unique.
recordNumber	An identifier given to the Occurrence at the time it was recorded. Often serves as a link between field notes and an Occurrence record, such as a specimen collector's number.
recordedBy	A list (concatenated and separated) of names of people, groups or organisations responsible for recording the original Occurrence. The primary collector or observer, especially one who applies a personal identifier (recordNumber), should be listed first.
eventDate	The date-time or interval during which an Event occurred. For occurrences, this is the date-time when the event was recorded. Not suitable for a time in a geological context.
year	The four-digit year in which the Event occurred, according to the Common Era Calendar.
month	The integer month in which the Event occurred.
day	The integer day of the month on which the Event occurred.
country	The name of the country or major administrative unit in which the Location occurs.
countryCode	The standard code for the country in which the Location occurs.
stateProvince	The name of the next smaller administrative region than country (state, province, canton, department, region etc.) in which the Location occurs.
county	The full, unabbreviated name of the next smaller administrative region than stateProvince (county, shire, department etc.) in which the Location occurs.
locality	The specific description of the place. Less specific geographic information can be provided in other geographic terms (higherGeography, continent, country, stateProvince, county, municipality, waterBody, island, islandGroup). This term may contain information modified from the original to correct perceived errors or standardise the description.
decimalLatitude	The geographic latitude (in decimal degrees, using the spatial reference system given in geodeticDatum) of the geographic centre of a Location. Positive values are north of the Equator, negative values are south of it. Legal values lie between -90 and 90, inclusive.
decimalLongitude	The geographic longitude (in decimal degrees, using the spatial reference system given in geodeticDatum) of the geographic centre of a Location. Positive values are east of the Greenwich Meridian, negative values are west of it. Legal values lie between -180 and 180, inclusive.
geodeticDatum	The ellipsoid, geodetic datum or spatial reference system (SRS) upon which the geographic coordinates given in decimalLatitude and decimalLongitude are based.
coordinateUncertaintyInMetres	The horizontal distance (in metres) from the given decimalLatitude and decimalLongitude describing the smallest circle containing the whole of the Location. Leave the value empty if the uncertainty is unknown, cannot be estimated or is not applicable (because there are no coordinates). Zero is not a valid value for this term.
georeferencedBy	A list (concatenated and separated) of names of people, groups or organisations who determined the georeference (spatial representation) for the Location.
identifiedBy	A list (concatenated and separated) of names of people, groups or organisations who assigned the Taxon to the subject.
typeStatus	A nomenclatural type (type status, typified scientific name, publication) applied to the subject.
scientificName	The full scientific name, with authorship and date information, if known. When forming part of an Identification, this should be the name in lowest level taxonomic rank that can be determined. This term should not contain identification qualifications, which should instead be supplied in the IdentificationQualifier term.
kingdom	The full scientific name of the kingdom in which the taxon is classified.
phylum	The full scientific name of the phylum or division in which the taxon is classified.
class	The full scientific name of the class in which the taxon is classified.
order	The full scientific name of the order in which the taxon is classified.
family	The full scientific name of the family in which the taxon is classified.
genus	The full scientific name of the genus in which the taxon is classified.
specificEpithet	The name of the first or species epithet of the scientificName.
infraspecificEpithet	The name of the lowest or terminal infraspecific epithet of the scientificName, excluding any rank designation.
taxonRank	The taxonomic rank of the most specific name in the scientificName.
scientificNameAuthorship	The authorship information for the scientificName formatted according to the conventions of the applicable nomenclaturalCode.
vernacularName	A common or vernacular name.
nomenclaturalCode	The nomenclatural code (or codes in the case of an ambiregnal name) under which the scientificName is constructed.
taxonomicStatus	The status of the use of the scientificName as a label for a taxon. Requires taxonomic opinion to define the scope of a taxon. Rules of priority then are used to define the taxonomic status of the nomenclature contained in that scope, combined with the experts opinion. It must be linked to a specific taxonomic reference that defines the concept.
acceptedNameUsage	The full name, with authorship and date information, if known, of the current botanical taxon.

## Additional information

### Biographical notes

The French missionary and botanist Fr. Urbain Jean Faurie (1847–1915) and Emile Joseph Taquet (1873–1952) greatly contributed to the understanding of the vascular flora on the Korean Peninsula and Quelpaert Island in the early 1900s. Taquet was born in Hecq, Quesnoy in France and was ordained when he was 24. As a local catholic priest and a field collaborator with Faurie, he made extensive collections of vascular plants on Island of Quelpaert from 1907 to 1915. Taquet stopped collecting plants when missionary Faurie died in Taiwan in 1915. In 1952, Taquet passed away at St Justin Catholic Seminary in Daegu, Korea. The major portions of their collected specimens are housed at E, P, TI and KYO. After the distribution to European and Japanese herbaria, many botanists have studied Faurie’s and Taquet’s collections: E. Hackel, A. E. Finet, P. C. Tsoong, H. Wolff, G. Koidzumi (Koidzumi 1943), B. Koehne, H. Boissieu, G. Hieronymus, H. Christ (Christ 1908), P. H. Lecomte, E. Palla, W. Becker, E. Hackel, A. Rehder, J. Ohwi, H. Léveillé (Léveillé 1907, Léveillé 1908a, Léveillé 1908b, Léveillé 1908c, Léveillé 1908d, Léveillé 1909, Léveillé 1910a, Léveillé 1910b, Léveillé 1910c), T. Nakai (Nakai 1909, Nakai 1911), G. Kükenthal and R. Kunth. Several specialists studied particular groups, such as ferns, sedges and trees, as well as specific genera.

## Figures and Tables

**Table 1.** While most South Korean place names are derived from words in the Chinese character, Japanese botanists transliterated these place names into the Japanese pronunciation.

**English names**	**Japanese exonyms**	**Endonyms**	**Korean word**
Mountain Hwa	Kazan	Hwasan	화산
Mountain Geumgang	Konggo-san	Geumgangsan	금강산
Yeongheung	Eiko	Yeongheungeup	영흥읍
Wonsan city	Genzan	Wonsansi	원산시
Gwaneum peak	Kannombo	Gwameumbong	관음봉
Pyohun temple	Hyokunji	Pyohunsa	표훈사
